# Antifibrotic Effect of *Bletilla striata* Polysaccharide-Resveratrol-Impregnated Dual-Layer Carboxymethyl Cellulose-Based Sponge for The Prevention of Epidural Fibrosis after Laminectomy

**DOI:** 10.3390/polym13132129

**Published:** 2021-06-29

**Authors:** Hsuan-Yu Chen, Tzu-Chieh Lin, Chih-Yung Chiang, Shiuan-Li Wey, Feng-Huei Lin, Kai-Chiang Yang, Chih-Hao Chang, Ming-Hsiao Hu

**Affiliations:** 1 Department of Biomedical Engineering, National Taiwan University, Taipei 106216, Taiwan; hychen83@gmail.com (H.-Y.C.); jacklin7412@gmail.com (T.-C.L.); okay0506@gmail.com (C.-Y.C.); double@ntu.edu.tw (F.-H.L.); 2 Department of Orthopedics, National Taiwan University College of Medicine and National Taiwan University Hospital, Taipei 100225, Taiwan; 3 Department of Orthopedics, National Taiwan University HsinChu Hospital, HsinChu 300016, Taiwan; 4 Department of Orthopedics, En Chu Kong Hospital, San-Shia, New Taipei City 23742, Taiwan; 5 Department of Pathology, Hsinchu Mackay Memorial Hospital, HsinChu City 30071, Taiwan; Shiuanliwey@gmail.com; 6 Department of Dental Technology, College of Oral Medicine, Taipei Medical University, Taipei 11031, Taiwan; pumpkin@tmu.edu.tw; 7 Department of Orthopedics, National Taiwan University Hospital, Jin-Shan Branch, New Taipei City 20844, Taiwan

**Keywords:** epidural fibrosis, laminectomy, *Bletilla striata*, carboxymethyl cellulose, resveratrol

## Abstract

The application of antifibrotic materials can alleviate epidural fibrosis by restricting excessive fibroblast proliferation and mitigating scar tissue formation. Here, a biodegradable carboxymethyl cellulose (CMC)-Bletilla striata polysaccharide (BSP)-resveratrol (RES) sponge was fabricated to inhibit scar tissue formation post laminectomy surgery. Fibroblasts NIH/3T3, myoblasts C2C12, neural cells PC-12, and Schwann cells RSC96 were used to evaluate the in vitro cytocompatibility. Laminectomies on 10 Sprague–Dawley rats with/without the application of the CMC-BSP-RES sponge were performed. The severity of adhesion between the dura mater and formed scar tissue was qualitatively scored. All cell lines exhibited good viability with no significant difference in cytotoxicity when cultured with variable extractions of the CMC-BSP-RES sponge. S100a4 and P4hb expressions were downregulated in NIH/3T3 cultured in the CMC-BSP-RES sponge, implying that this sponge potentially inhibits fibroblast activity. No post-operative shrinkage or dura mater expansion along the surgical site was detected. The peel-off tests revealed that the tenacity of adhesion de-creased. Histopathological examinations verified that the average number of fibroblasts in the CMC-BSP-RES group considerably decreased. The CMC-BSP-RES sponge is a biocompatible and effective material for alleviating post-operative epidural fibrosis and mitigating fibroblast expression following laminectomy.

## 1. Introduction

Laminectomy is a surgical procedure typically employed in the treatment of numerous spinal disorders such as lumbar disk herniation, spinal stenosis, and tumor excision [1]. Although this treatment is generally effective and relieves neural compression, 8–40% of patients suffer from failed back surgery syndrome (FBSS), with 4–9% undergoing revision surgery [2,3].

The process of scar tissue formation can be divided into three phases. During the first phase, hemostasis and coagulation, along with chemokine release, are observed in the first 3–5 days after surgery [4]. In the second phase, which lasts approximately 2–3 weeks, fibroblast proliferation and differentiation, granulation tissue formation, and collagenous fiber secretion in the epidural defect space begin to manifest [5]. In the third phase, which can last from months to years, scar connective tissue begins to reconstruct and transform around the defect lesion [6]. The above-described exaggerated and dysregulated “healing” mechanisms, as a response to trauma, culminate in local acute inflammations, extracellular matrix deposition, fibrosis, and neo-angiogenesis [7]. Several factors, such as individual variability, wound healing process, hematoma, soft tissue amount, and bone traumatization are involved in the process, and they have important roles in the pathogenesis of epidural fibrosis. The aforementioned adhesions potentially contribute to 20–36% of the reported FBSS cases, by inducing nerve irritation, dura sac compression, and back pain (especially with movements including the flexion and/or extension of the back) [8] and increase the technical difficulty and risks associated with revision surgery. The goal of treatment is to prevent epidural fibrosis formation because the surgical removal of fibrous tissue is often associated with poor results. Furthermore, the number of patients suffering from epidural fibrosis is increasing, especially given the substantially higher rates of spine surgery in the past few decades [9].

Currently, the prevention of epidural adhesion remains a major challenge in the field of spinal surgery. Various clinical strategies have been introduced to attenuate epidural fibrosis, including minimal invasive surgery, radiotherapy, drug treatment, and material placement. Drugs including mitomycin C [10] and suramin [11] have also been employed, along with non-steroidal anti-inflammatory drugs [12] and certain bioactive compounds found in traditional Chinese plants (such as salvianolic acid B [13] and icariin [14]). Autologous fat grafting is the most commonly employed technique for preventing epidural fibrosis; however, the clinical findings are controversial as fat grafts are susceptible to degradation due to atrophy and necrosis [15], which can potentially induce the cauda equina syndrome because of compression of the dura sac [16].

With the advancement of material science and bioengineering, novel biodegradable polymeric biomaterials, capable of providing a mechanical barrier to decrease the adhesion strength between the scar tissue and dura mater, have gained substantial scientific interest. Examples of such biomaterials are space-occupying agents, including hyaluronic acid-derived gel [17], chitosan-dextran gel [18], carboxymethyl cellulose (CMC), and polyethylene oxide [19,20]. However, strategies based on these materials continue to present severe hindrances, such as clinically unsatisfactory applicability, inhibiting dural healing from pinhole lesion, and exacerbating cerebral spinal fluid leakage ([21]; therefore, developing advanced anti-adhesive biomaterials remains challenging.

CMC, biocompatible polysaccharide, possess improved degradable absorption and natural integration within host tissues. Biochemically, CMC is a high molecular weight polymer that is water soluble, heat stable, and available in various molecular weights and viscosities that has been experimentally confirmed to be a suitable drug release carrier and decrease post-surgical adhesions in several field [17,19,20]. Resveratrol (3,5,4′-o-trihydroxystilbene) (RES) exhibits antioxidant, anti-inflammatory, antifibrotic, and antiproliferative properties [22,23]; RES is extremely unstable in water, but strong stabilizing effect in aqueous media is noticed when RES is combined with CM-glucan matrices. The water-soluble *Bletilla striata* polysaccharide (BSP) is isolated from the terrestrial orchid *Bletilla striata*, mostly found in East Asian countries [24,25]. Furthermore, it has been confirmed to exhibit anti-inflammatory, antifibrotic, and antioxidant properties [26,27]. In addition, BSP contributes to the process of wound healing by controlling pro-inflammatory factors, thereby minimizing inflammation at the site [28]. Studies have also indicated that BSP successfully downregulated the mRNA expression of transforming growth factor-beta receptor type 1 (*TGF-β RI*), *TGF-β RII*, and alpha-smooth muscle actin (*α-SMA*) in human mesangial cells, thereby effectively reducing TGF-beta signaling pathways [26,29].

**Figure 1:**
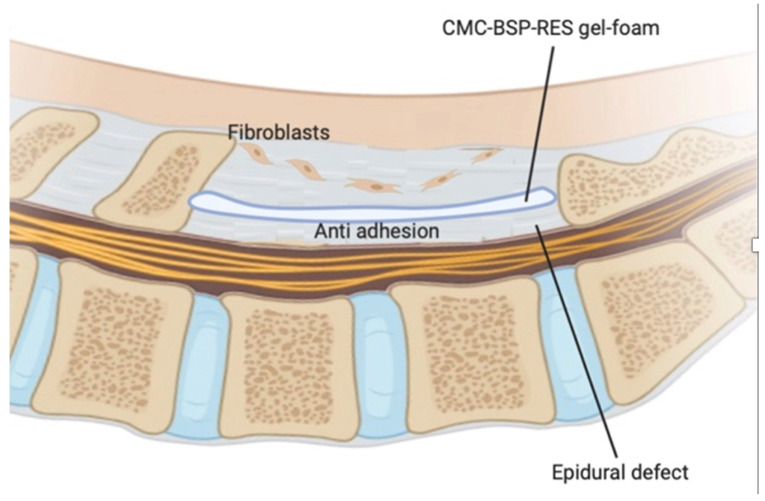
Schematic of the dual-layer CMC-BSP-RES sponge proposed in this study to effectively block invasive fibroblasts and reduce subsequent scar development in epidural defects and to promote post-wound regeneration.

**Figure 2:**
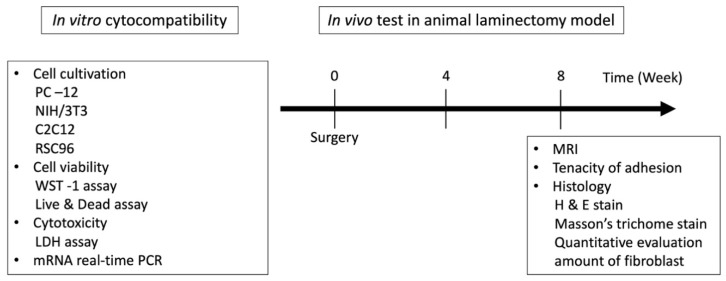
Experimental setup. The cytocompatibility of the sponge was tested, and its effect on epidural fibrosis in a rat model after laminectomy was evaluated.

Based on available literature, we aimed to fabricate a BSP-RES-impregnated dual layer CMC-based sponge for preventing scar tissue formation in the epidural space following laminectomy surgery. in this study, we fabricated BSP-RES-impregnated dual layer CMC-based sponge; the schema of study design is depicted in Figure 1. To evaluate the cytocompatibility of the CMC-BSP-RES sponge, in vitro cell cultivation experiments and characterization of sponge with cell viability and cytotoxicity assays were performed. To investigate the efficacy of the proposed material in preventing epidural fibrosis, the CMC-BSP-RES sponge was evaluated by quantitative real-time polymerase chain reaction mRNA gene expression in vitro and in vivo experiments using a rat laminectomy model. The severity of scarring and adhesion in all treatment groups was analyzed through magnetic resonance imaging (MRI), gross inspection, and histologic examination. The experimental setup is illustrated in Figure 2.

## 2. Materials and Methods

### 2.1. Materials and Reagents

RES (Sigma, R5010, Saint Louis, MO, USA) and CMC (Sigma, C4888, Saint Louis, MO, USA) used in this study were purchased from Sigma-Aldrich (St. Louis, MI, USA). *Bletilla striata* was purchased from Sheng Chang Company (Taoyuan, Taiwan), and fetal bovine serum (FBS) was purchased from Gibco BRL (Gaithersburg, MD, USA). 

### 2.2. Bletilla striata Polysaccharide Extraction

The extraction of BSP from *B. striata* has been described in our previous study [30]. In brief, dried *B. striata* were ground, dispersed in double-distilled water (ddH_2_O) at 80 °C for 4 h, and subsequently, filtered to remove impurities. The crude extracts were then subjected to chemical precipitation with 95% (*v*/*v*) ethanol solution overnight. After centrifugation, the obtained precipitates were dissolved in distilled water, and deproteinized by adding 1/3 volume of Sevage reagent (chloroform/n-butanol 4:1) with overnight stirring. This procedure was repeated 2–3 times. The resultant aqueous phase was dialyzed using a dialysis tube with a molecular weight cut-off (MWCO) of 3000–5000 Da and subsequently lyophilized to obtain BSP. Fourier-transform infrared (FTIR) spectroscopy and ^13^C and ^1^H nuclear magnetic resonance (NMR) spectroscopy were conducted to analyze the structural properties of the obtained polysaccharides [31].

### 2.3. Preparation of a Dual-Layer CMC-BSP-RES Sponge

**Figure 3:**
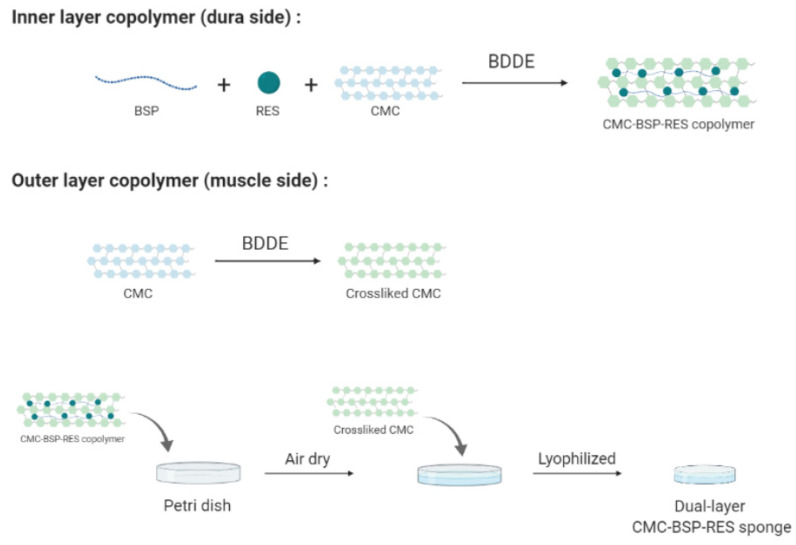
Schematic of the formation of a dual-layer CMC-BSP-RES sponge.

The dual-layer CMC-BSP-RES sponge was fabricated as shown in Figure 3. For the inner layer (in contact with the dura mater), 0.05 g of BSP and 114 mg of RES were dissolved in 100 mL of sodium hydroxide solution (1%) and stirred for 4 h at 22–26 °C to obtain a 0.05% BSP/5 µM RES solution. Subsequently, 3.5 g of CMC powder was dissolved in the BSP-RES mixed solution and stirred overnight at 4 °C. Thereafter, 0.1% (*v*/*v*) of 1,4-butanediol diglycidyl ether (BDDE) was added to the solution and stirred for 4 h at room temperature for crosslinking. Finally, the CMC-BSP-RES solution was dialyzed in ddH_2_O for 2 days (MWCO: 12,000–14,000). For the outer layer (in contact with muscle tissue), CMC solution (without BSP-RES) was prepared. CMC-BSP-RES solution (3 mL) was poured into a 35-mm Petri dish and air-dried for 1 day. The BSP-RES-free CMC solution was then added over the CMC-BSP-RES layer and freeze-dried to obtain the dual-layer sponge.

### 2.4. Cultivation of Cell Lines

Four different cell lines, NIH/3T3 fibroblasts (60008; BCRC, Hsinchu, Taiwan), C2C12 myoblasts (60083; BCRC), PC-12 neural cells (60048; BCRC), and RSC96 Schwann cells (60507; BCRC) were cultured to investigate the cytocompatibility of the CMC-BSP-RES sponge. High-glucose Dulbecco’s modified Eagle’s medium (DMEM) supplemented with 10% FBS, L-glutamine (0.584 g/L), and 1% antibiotic (10,000 units penicillin, 10,000 μg streptomycin, and 25 µg amphotericin B; 516104-M, Millipore) was used to culture NIH/3T3, RSC96, and C2C12 cells. PC-12 cells were cultured in 85% Roswell Park Memorial Institute (RPMI) 1640 medium, supplemented with 5% FBS, 1% antibiotic, l-glutamine, and 10% heat-inactivated horse serum. The culture flasks were maintained in an incubator at 37 °C in a humidified atmosphere with 5% CO_2_.

### 2.5. Cytocompatibility of the CMC-BSP-RES Sponge

The CMC-BSP-RES extraction medium was prepared according to ISO 10993-5 and used for cytocompatibility evaluations. In brief, the CMC/BSP/RES dual-layer sponge was immersed in serum-free culture medium of a series of weight ratios (10, 5, 1, 0.5, and 0.1 mg samples per mL culture medium) under mild shaking at 37 °C for 24 h. We seeded 2.5 × 10^3^ of C2C12 myoblasts and RSC96 Schwann cells, and 5 × 10^3^ of PC-12 neural cells and NIH/3T3 fibroblasts in a 96-well cell culture polystyrene plates at a density of 1 × 10^4^ cells/well; the cells were cultured in relevant culture media overnight [17]. Subsequently, the cells were cultured in the CMC-BSP-RES sponge extraction medium at 37 °C. Cells treated with medium containing 0.2 g/mL zinc diethyldithiocarbamate (ZDEC) were used as the positive control group. Cells treated with medium containing 0.2 g/mL aluminum oxide (Al_2_O_3_) were used as the negative control. Triton X-100 and serum-free medium were used for comparison. After 24 h of culture, the culture media were collected for other analyses, and cell viability was evaluated using water-soluble tetrazolium-1 (WST-1, Roche) reagent. The absorbance of the solution was measured using a spectrophotometer (reference wavelength = 650 nm) at 450 nm. The cytotoxicity of the CMC-BSP-RES sponge was evaluated using the lactate dehydrogenase (LDH) assay kit. The preserved culture media were reacted with the assay kit reagents for 30 min in the absence of light. Cell cytotoxicity was determined to be directly proportional to the LDH release rate, which was measured using a spectrophotometer at 490 nm.

Furthermore, the survival of cells cultured in different extraction media was determined using the live/dead double staining assay (R37601, LIVE/DEAD^®^ Cell Imaging Kit; Thermo Fisher Scientific, Waltham, MA, USA), and cell survival was observed by fluorescence microscopy.

### 2.6. mRNA Expression in Cells in the CMC-BSP-RES Sponge

We suspended 4 × 10^5^ NIH/3T3 fibroblasts and PC12 neural cells, and 2 × 10^5^ C2C12 myoblasts and RSC96 Schwann cells in 200 μL of culture media and seeded in a CMC-BSP-RES sponge using a micropipette. The cells in the inner layer of the dual-layer sponge were incubated in the 24-well culture plate for 30 min for cell adhesion, and finally, 1 mL of culture medium was added. The same quantity of cells was seeded to a 12-well culture plate and cultured in a standard medium as a reference.

After 1 week of culture, the total RNA of cells cultured in the dual-layer CMC-BSP-RES sponge was extracted and reverse transcribed into cDNA (MultiScribe™ Reverse Transcriptase, ThermoFisher). The cDNA was subjected to Assay-On-Demand™ Gene Expression probes with the TaqMan Universal PCR Master Mix in an ABI PRISM 7900 HT sequence detection system. For NIH/3T3 fibroblasts, the mRNA expression of S100 calcium-binding protein A4 (*S100a4*) (Mm00803372_g1) and beta-subunit of prolyl 4-hydroxylase (*P4hb*) (Mm01243188_m1) was determined. Myogenic factor 6 (*Myf6*) (Mm00435127_g1), myoblast determination protein 1 (*MyoD1*) (Mm01203489_g1), and paired box protein 7 (*Pax7*) (Mm01354484_m1) were analyzed in C2C12 myoblasts. *S100b* (Rn04219408_m1) expression was evaluated in PC12 neural cells and RSC96 Schwann cells. Glyceraldehyde-3-phosphate dehydrogenase (*GAPDH*) (Mm99999915_g1, Rn99999916_s1) was used as the house-keeping gene.

### 2.7. In Vivo Experiments

**Figure 4:**
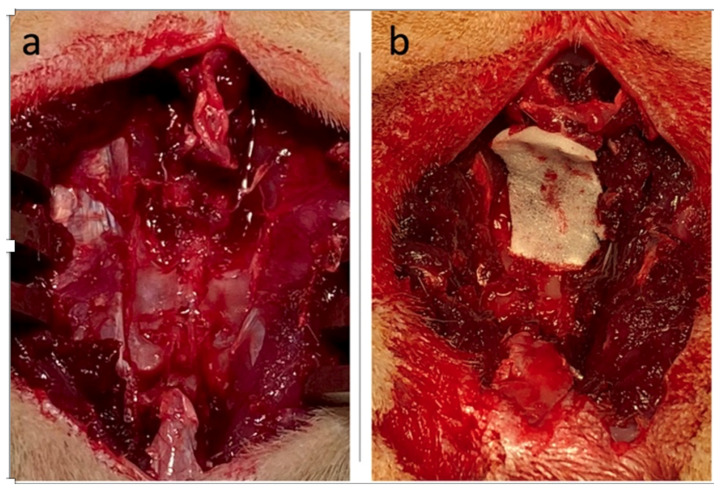
Dura theca and nerve root were covered with the inner layers of the CMC-BSP-RES sponge in the treatment group after laminectomy. (**a**) Control group; (**b**) treatment group.

The experimental protocol and retrieval process of animal tissues were approved by the National Taiwan University Hospital College of Medicine Institutional Animal Care and Use Committee (IACUC number 20200031). In total, 10 Sprague–Dawley (SD) rats (male, 10–11 weeks old) were obtained from the laboratory animal center of the National Taiwan University College of Medicine, Taipei, Taiwan. The surgical procedure was based on a previous study [17]. Concisely, the rats were fixed in prone position and subjected to intra-abdominal anesthesia with 30 mg/kg sodium pentobarbital injection. Next, two midline skin incisions were separately performed on the thoracic and lumbar spine regions. Using a microscope, the two laminectomy areas in the mid-thoracic and lumbar spine regions were randomly assigned as the treatment and control groups by the same surgeon. After performing laminectomy in the treatment group, the dura theca and nerve root were covered with the inner layers of the CMC-BSP-RES sponge (Figure 4). Finally, the wounds were closed through hemostatic control.

### 2.8. MRI Inspection and Peel-Off Testing

To measure the scar-adhesion operative field, we acquired T2-weighted 7T-MRI slices of the spinal cord in the transverse plane (TR = 3000 ms, TE = 26.7 ms, slice thickness = 1 mm) at 8 weeks after laminectomy. Subsequently, the rats were euthanized by intra-abdominal injection of high doses (75–100 mg/kg) of sodium pentobarbital, and then revision surgery was performed. The scar tissue was peeled off manually using a pair of forceps. During this process, a four-level (grades 0–3) qualitative assessment of the scar-adhesion strength was performed to evaluate the tenacity of adhesion between the scar tissue and dura mater. Grade 0 implied no substantial adhesion between the dura mater and fibrotic tissue; grade 1 implied slight adhesion, that is, fibrotic tissue could be easily detached from the dura mater without manual force; grade 2 implied moderate adhesion, that is, fibrotic tissue could be detached from the dura mater by applying moderate traction; grade 3 implied tenacious adhesion, that is, fibrotic tissue could be detached from the dura mater only by sharp dissection [32].

### 2.9. Preparation of Specimens and Histopathological Examination

After inspecting MRI slices, the rats were sacrificed and the entire thoracic and lumbar spine regions were removed. The thoracic/lumbar spine regions were fixed in 10% formalin containing a neutral buffer for 2 days and subsequently immersed in a decalcification solution (0.5 M aluminum chloride, 8.5% HCl, and 5% formic acid) for an additional 3 days. Routine tissue processing was performed on a 2 mm-thick specimens (transverse to the spinal canal at the laminectomy site); serial sections of 4-µm paraffin-embedded block specimens were stained with hematoxylin and eosin (H&E) and Masson’s trichrome kits using appropriate kits (Polysciences, Warrington, PA, USA). The dura adhesion of the specimens was evaluated under a microscope, according to the criteria proposed by He et al. [33]. The density of the fibroblasts were counted in each field at 40× magnification, with grade I defined as less than 100 fibroblasts/field, grade II as 100–150 fibroblasts/field, and grade III as above 150 fibroblasts/field [34].

### 2.10. Statistical Analysis

The data were statistically evaluated using a single factor one-way analysis of variance (ANOVA) and all data are presented as standard errors of the mean (SEM). SPSS 19.0 software was used to perform the statistical analyses, and a difference was considered statistically significant at *p*-value less than 0.05.

## 3. Results

### 3.1. Influence of the CMC-BSP-RES Sponge on Mitochondrial Activity

**Figure 5:**
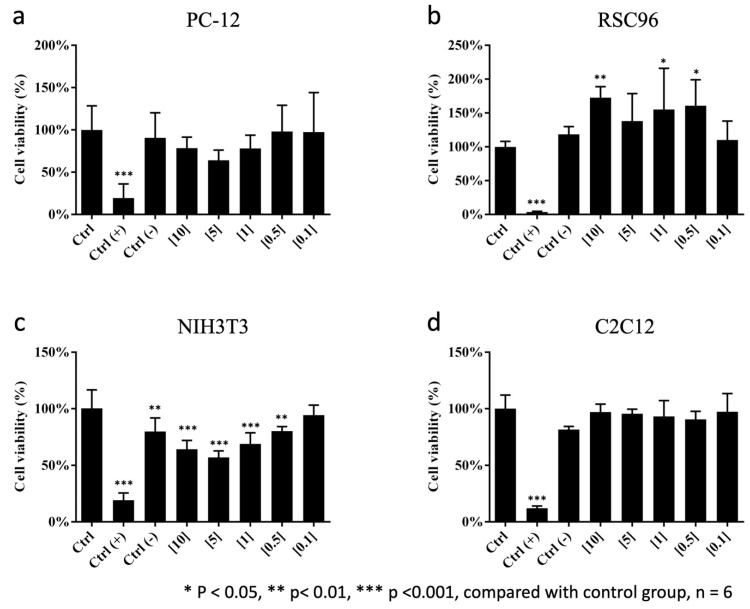
Viability of cells cultured in extraction media of CMC/BSP (10, 5, 1, 0.5, and 0.1 mg/mL)/RES dual-layer sponges. (**a**) PC12; (**b**) RSC96; (**c**) NIH/3T3; (**d**) C2C12.

The viability of the four cell lines cultured in the CMC/BSP/RES sponge extraction media at various weight ratios was evaluated using the WST-1 regent. The PC-12 neural cells demonstrated good viability (>70%) when cultured in CMC-BSP-RES sponge extractions at all concentrations (Figure 5a). Similarly, the viability of C2C12 myoblasts did not significantly change when cultured in the extractions (Figure 5d). Interestingly, the viability of RSC96 Schwann cells was significantly increased (>100%) after culture in 10, 1, and 0.5 mg/mL CMC-BSP-RES sponge extraction media (Figure 5b). On the contrary, PC-12 neural cells and NIH/3T3 fibroblasts presented good viability only when cultured in 0.1 mg/mL CMC-BSP-RES sponge extraction media (Figure 5**c**).

### 3.2. LDH Cytotoxicity Assay and Cell Survival

**Figure 6:**
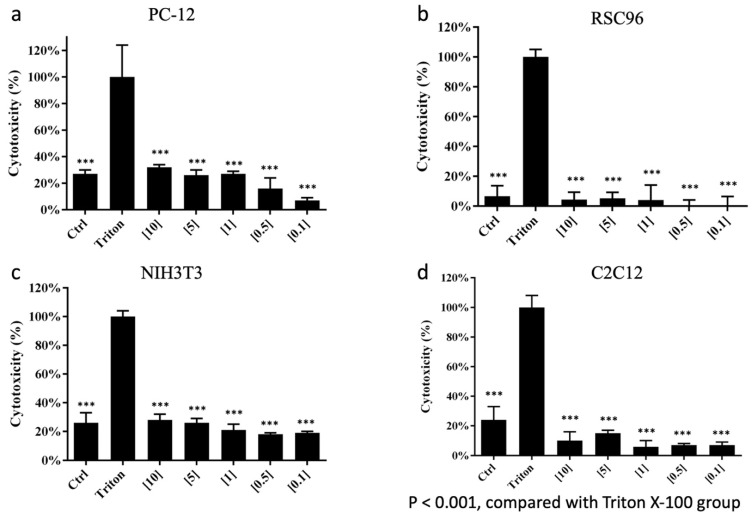
(**a**,**c**) Higher cytotoxicity was observed in PC-12 and NIH/3T3 cell lines after culture in high-concentration of CMC/BSP (10, 5, and 1 mg/mL)/RES sponge (cytotoxicity > 20%) using the LDH assay kit. (**b**,**d**) There were no significant differences between RSC96 and C2C12 cell lines after culture in CMC-BSP-RES sponges at different concentrations. ***: *p* < 0.001.

**Figure 7:**
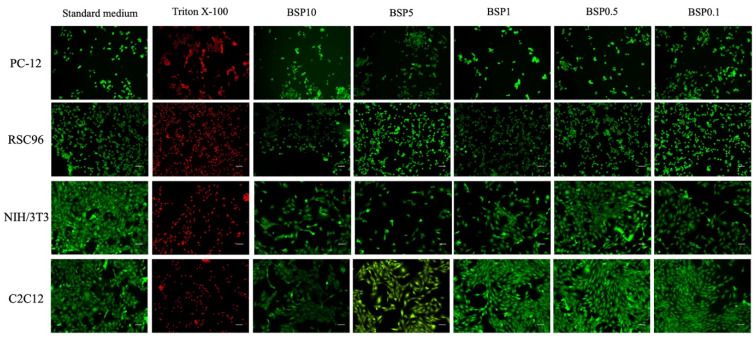
Similar cell counts and survival rates were observed between fluorescence-based live/dead staining and LDH tests under different CMC-BSP-RES sponge concentrations. PC12 neural cells; RSC96 Schwann cells; NIH/3T3 fibroblasts; and C2C12 myoblasts.

No significant differences were found in RSC96 Schwann cells and C2C12 myoblasts when cultured in different extractions of the CMC-BSP-RES sponge (Figure 6b,d). Relatively high cytotoxicity (>20%) was observed in PC-12 and NIH/3T3 cell lines according to the qualitative morphological grading of cytotoxicity, after culture in high-concentration CMC/BSP (10, 5, and 1 mg/mL)/RES sponges (Figure 6a,c). Interestingly, the fluorescence-based live/dead staining assay with different concentrations of CMC-BSP-RES sponges presented comparable cell counts and survival rates with the LDH test results (Figure 7). Based on the above results, 0.1 mg/mL CMC-BSP-RES sponge was selected for use in the subsequent experiments.

### 3.3. mRNA Expression Analysis by RT-qPCR

**Figure 8:**
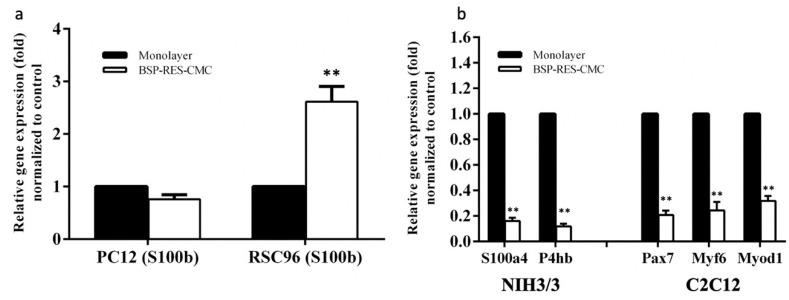
Gene expression in (**a**) PC12 and RSC96; (**b**) NIH/3T3 and C2C12 cell lines cultured in monolayers or CMC-BSP-RES sponges. **: *p* < 0.01, compared with monolayer.

When PC-12 neural cells were cultured in the CMC-BSP-RES dual-layer sponges, there was mild downregulation of *S100b* mRNA relative to that in the monolayer culture group; no other significant differences were noticed between these groups. In contrast, *S100b* expression was significantly upregulated to that in the monolayer culture group when RSC96 Schwann cells were cultured in the CMC-BSP-RES dual-layer sponge (*p* < 0.01). The *S100a4* (*p* < 0.01) and *P4hb* mRNA expressions (*p* < 0.01) in NIH/3T3 fibroblasts cultured in the CMC-BSP-RES dual-layer sponge were significantly downregulated to that in the monolayer culture group. Similarly, the downregulation of the mRNA expression of *Pax7* (*p* < 0.01), *Myf6* (*p* < 0.01), and *Myod1* (*p* < 0.01) was observed when C2C12 myoblasts were cultured in the CMC-BSP-RES sponge (Figure 8).

### 3.4. MRI, Peel-Off Testing, and Histological Analysis

**Figure 9:**
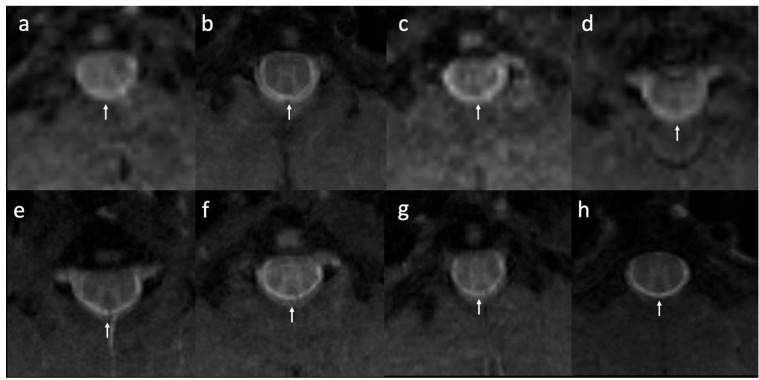
T2-weighted MRI slices. No post-operative shrinkage or dura mater expansion was observed along the surgical site. The thickness of the epidural fibrotic tissue was relatively thinner in the treatment group and thicker in the control group. (**a**–**d**) control group; (**e**–**h**) treatment group (white arrow: epidural fibrosis).

**Figure 10:**
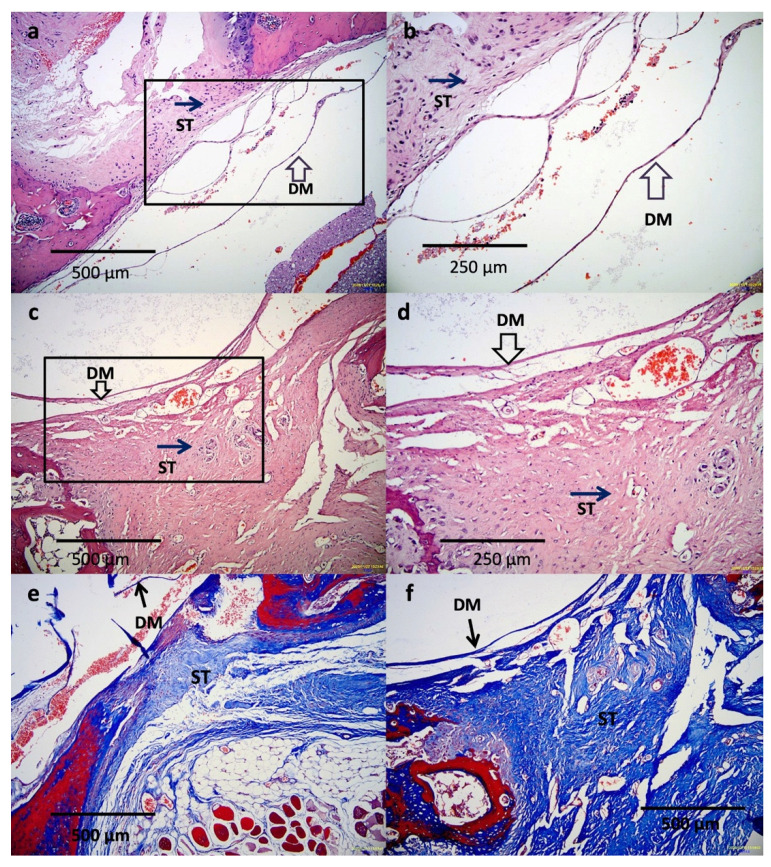
Histology results at 8 weeks post-laminectomy. (**a**) 100× and (**b**) 200× H&E-stained image of a specimen of the treatment group. There were only thin fibrotic bands between the scar tissue and dura mater. (**c**) 100× and (**d**) 200× H&E-stained image of a specimen of the control group. Large-area fibrotic adhesion in the epidural defect was observed. (**e**) 100× Masson’s trichrome-stained image of a specimen of the treatment group. There were increased collagen fibers compared with (**f**) 100× image of the control group. (ST: scar tissue, DM: dura mater).

Signal and diameter changes in the sample regions were evaluated using MRI sequential analysis; there was no post-operative shrinkage or dura mater expansion along the surgical site. The results also showed that the thickness of the epidural fibrotic tissue was confirmed to be relatively thinner in the treatment group and thicker in the control group (Figure 9). Among the 10 rats used in our study, two suffered from post-operative paralysis, and they were excluded from the analysis. The remaining 10 rats presented good wound healing, without signs of swelling, inflammatory discharge, or infection at 8 weeks after surgery; they were subjected to peel-off test and histological analysis. No residual sponge was detected in the treatment group; however, one rat exhibited a disruption of the dura mater in the control region during the peeling-off test. According to the scar scoring system results, the mean grade was 0.8 ± 0.36 in the treatment group and 2.1 ± 0.49 in the control group. As expected, the tenacity of adhesion between the fibrotic tissue and dura mater was significantly higher in the control group, as determined using the peel-off test (*p* < 0.05). H&E staining revealed that hyperemia and inflammatory reactions in the rats treated with the CMC-BSP-RES sponges were substantially alleviated and only thin fibrotic bands were formed between the scar tissue and dura mater (Figure 10a,b). On the contrary, we observed large-area fibrotic adhesion in the epidural defect of the control group, which additionally exhibited admixed lymphocytes and angiogenesis in week 8 histology results (Figure 10c,d). The thickness of the collagen layer was examined by Masson’s trichrome staining. The collagen fibers significantly increased in terms of thickness in the epidural space of the control group (Figure 10e,f).

**Figure 11:**
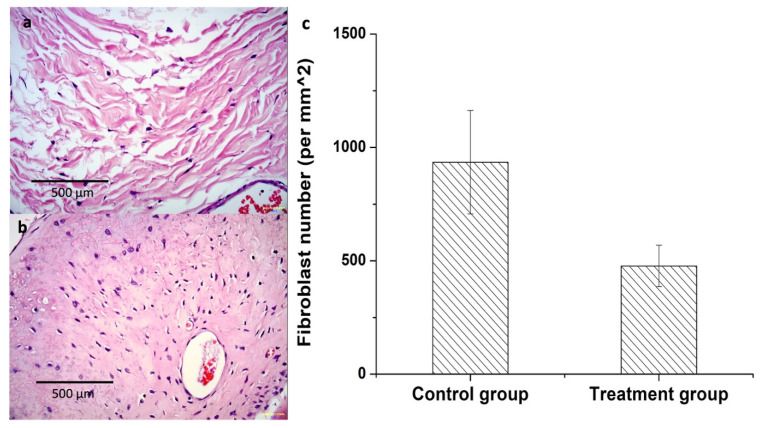
Histology results at 8 weeks post-laminectomy. 400× H&E-stained image of a specimen. (**a**) Treatment group showed significantly reduced number of fibroblasts in epidural defect in comparison with (**b**) the control group. (**c**) The average fibroblast count was 477 ± 91 per mm^2^ in the treatment group and 935 ± 228 per mm^2^ in the control group 8 weeks postoperatively (n = 10, mean ± SD).

The number of fibroblasts was significantly lower in the CMC-BSP-RES group, indicating that the CMC-BSP-RES sponge effectively alleviated the inflammatory reaction of epidural fibrosis in the animal models (Figure 11a,b). The average fibroblast number was 477 ± 91 per mm^2^ in the CMC-BSP-RES group and 935 ± 228 per mm^2^ in the control group at 8 weeks postoperatively (*p* < 0.05) (Figure 11c).

## 4. Discussion

Spinal laminectomy is an effective surgical technique capable of relieving neural compression in patients suffering from myelopathy or radiculopathy. However, the development of post-operative fibrotic adhesions within the epidural space is a clinical scenario of the post-surgery tissue healing process. An ideal barrier to prevent or reduce adhesions between dura sac and paravertebral soft tissue should mimic the physiological features of original epidural fat. Barrier materials are meant to avoid the formation of scar tissue between them and decrease the dead space. The application of antifibrotic materials can alleviate epidural fibrosis by restricting the excessive proliferation of fibroblasts and by mitigating scar tissue formation. Natural polymeric materials can be ideal biomaterials as physical barriers against fibrotic adhesion, and they eliminate the abovementioned complications post laminectomy. Accordingly, BSP-RES-impregnated dual-layer CMC-based sponge was developed to prevent epidural fibrosis.

RES is a polyphenol known to be present in grapes, red wine, and medicinal plants [35]. However, there is a lack of studies exploring the potential of RES in preventing scar adhesions in animal models. Recently, Sun et al. (2014) demonstrated the effectiveness of RES in downregulating inflammatory factors (such as TGF-β1 and IL-6) in rat models following laminectomy, thereby minimizing the severity of epidural fibrosis. However, the major concerns in applying RES in humans are its limited bioavailability and the lack of sufficient scientific findings to support further clinical evaluation and utilization of RES [36]. RES, which is susceptible to degradation when solubilized in water (as a result of oxidation), is an extremely unstable molecule. When RES molecules are combined with CM-glucan matrixes, they are endowed with a strong stabilizing effect in aqueous media, thus extending RES stability in water up to 12 months [37]. This phenomenon could be attributed to the exertion of a physical barrier from the CM-glucan matrixes through the encapsulation of RES molecules or a weak electrostatic interaction between the two compounds. Similarly, *B. striata* is a hemostatic medicinal plant that has been used for almost 2000 years in China. Recently, Liu et al. (2019) demonstrated that BSP treatment could effectively reduce the inflammatory reactions preventing intestinal adhesion after operation, and mitigate the generation of inhibitory substances, such as TGF-*β* RI, TGF-β RII, and α-SMA, which induced cell differentiation into fibroblasts. BSP polymer comprises glucopyranose and mannopyranose, and studies have hypothesized its effect persists even when rapidly dissolved or released in an aqueous environment in vivo during degradation [24]. Although we do not know whether this effect results from the polymer or monomer, BSP significantly improves the efficacy in epidural fibrosis prevention in animal models when rapidly released in the initial stage, such as nerve regeneration [38]. Therefore, a RES- and BSP-impregnated dual-layer CMC sponge was fabricated.

Space-occupying biomaterials, including hyaluronic acid-derived gel and chitosan-dextran gel, might unmask unrecognized durotomies by inhibiting dural healing or exacerbating cerebral spinal fluid leakage. In addition, chitosan is known to inhibit fibrosis by inhibiting TGF-β signaling [39]. Hyaluronic acid is also known to possess anti-inflammatory effects [40]. The antifibrotic property of these biomaterials may inhibit dural healing, whereas pinhole tears cannot undergo normal healing, or these materials may prevent clot formation and separate the paraspinous muscle from lesion dura. The dual-layer sponge designed in this study effectively blocked invasive fibroblasts and saturated the surgical site when applied post surgery, thus acting as a biocompatible physical barrier and inhibiting fibrosis from exaggerated and dysregulated “healing” mechanisms. Two types of precursor solutions were utilized in the fabrication of the dual-layered sponge. The inner layer of the CMC-BSP-RES sponge was anticipated to limit epidural fibrosis formation between the soft tissue and dura mater, by efficiently downregulating fibroblast cells in the in vitro study. The outer layer contained only CMC and could act as an adhesive and healing factor for normal scar formation. The results of the WST-1 assay indicated that NIH/3T3 fibroblasts were more sensitive to BSP concentration than PC-12, RSC96, and C2C12 cells, because a significant decrease in the viability of former cells was observed when BSP concentration exceeded 0.5 mg/mL. The LDH assay results revealed that less than 20% of the cells were round, devoid of intra-cytoplasmic granules, and exhibited cell lysis in PC-12 and NIH/3T3 cells when the BSP concentration was within 0.1 mg/mL. This agrees with the findings of the live/dead staining test.

*S100a4*, a member of the S100 family secreted by stromal cells, and *P4hb*, a fibroblast marker, have been shown to contribute to fibrotic disease [41]. According to the PCR results, both genes were downregulated in NIH/3T3 fibroblasts when cultured in 0.1 mg/mL CMC-BSP-RES sponge. Similarly, *MyoD1*, which plays a major role in muscle growth and development, *Pax7*, a transcription factor essential for the renewal and maintenance of muscle stem cells, and *Myf6*, which is highly expressed in differentiated skeletal muscle, were downregulated in C2C12 cells [42]. Contrarily, *S100b*, a small EF-hand calcium- and zinc-binding protein, which is known to suppress Schwann cell proliferation but promote myelination, was upregulated in RSC96 cells [43]. All these findings support the application of the dual-layer CMC-BSP-RES sponge in the prevention of epidural fibrosis.

The alleviating effects of BSP on fibroblast migration and inflammatory response exhibited a dose-dependent behavior, at concentrations above 1 mg/mL exerting negative effects in the in vitro study. No clinical signs of toxicity manifested during our observations, with all rats preserving regular vitality in all experimental stages. The MRI analysis, which is regarded as the gold standard for evaluating FBSS severity, confirmed the healthy and well-healed paraspinal muscular structure of the animal models. After performing revision surgery, the peel-off test results indicated significantly decreased adhesion tenacity in the treatment groups. A significantly reduced number of fibroblasts was detected in the treatment group, further implying the desirable effect of the CMC-BSP-RES sponge. The sponge was also confirmed to reduce post-operative epidural fibrosis to a limited extent. The primary disadvantage of the sponge is that it can potentially form a hematoma after blood absorption, which transforms into a scar tissue with epidural fibrosis, and oppresses the nerve root or dura sac. Achieving optimal hemostasis during surgery is essential for promoting a balanced healing process and ensure that no post-operative shrinkage or expansion of the dura mater occurs along the surgical site. The latter can be corroborated by MRI observation. Furthermore, the sponge is characterized by a short persistent time and poor mechanical strength. The degradation time of sponge in the treatment group was measured to be approximately 14 days post laminectomy [44], suggesting its concurrence with the timeframe of the wound healing process, which is critical for controlling the first and second phases of epidural fibrosis development. However, previous studies showed the highest activity of fibrous connective tissue ingrowth may last for 4–6 weeks. Future works including modifying material components to elongate the degradation time should be addressed. The development and approval of anti-adhesive biodegradable polymeric materials has economical potential in medical market on the basis of the growing need for preventing epidural fibrosis formation leading to failed back surgery syndrome in clinical practice. One of the challenges is evaluation of clinical efficacy in lack of specimen since nerve biopsy is pretty well impossible. Regardlessly, the results of our study demonstrated the potential of the CMC-BSP-RES sponge in clinical application.

## 5. Conclusions

To the best of our knowledge, this is the first paper to report the application of the CMC-BSP-RES sponge in antifibrotic tissue engineering and its promising behavior as a composite immune-modulatory barrier agent possessing hemostatic, antifibrotic, and anti-adhesion properties. We believe that the results of this study highlight a new and effective treatment strategy for the prevention of post-laminectomy epidural fibrosis formation using biomaterials.
